# Latent profile analysis of fertility intention among women of reproductive age

**DOI:** 10.3389/frph.2026.1758039

**Published:** 2026-02-10

**Authors:** Miaomiao Chen, Shailing Ma, Xiaohui Liu, Lijun Wang, Yingjie Zheng, Jiajia Lai, Jing Li, Yijia Qi

**Affiliations:** School of Nursing, Ningxia Medical University, Yinchuan, Ningxia, China

**Keywords:** fertility intention, influencing factors, latent profile, reproductive age, women

## Abstract

**Background:**

China's total fertility rate has reached a critically low level, dropping to approximately 1.0 by the end of 2023which is significantly below the population replacement level of 1.5. This decline reflects a marked reduction in fertility intention among reproductive-aged women, exacerbating population aging and threatening long-term labor supply and social sustainability. Despite policy adjustments and governmental support initiatives, intended outcomes have not been realized. Current literature largely focuses on isolated determinants of fertility intention, overlooking heterogeneity within the population. Moreover, the pathways through which psychosocial factors operate across different subgroups remain poorly understood.

**Methods:**

Data for this study were derived from the 2021 Psychological and Behavioral Investigation of Chinese Residents (PBICR 2021), a nationally representative cross-sectional survey. Latent profile analysis (LPA) was employed to identify subtypes of fertility intention among reproductive-aged women, followed by multinomial logistic regression, which examined factors associated with different profiles.

**Results:**

Among 2,973 reproductive-aged female participants, three distinct fertility intention profiles were identified via latent profile analysis: the Fertility Intention Decline Group (25.1%), the Low Fertility Intention Group (51.3%), and the High Fertility Intention Group (23.6%). Multinomial logistic regression analysis revealed that, compared with the Fertility Intention Decline Group, the Low Fertility Intention Group was significantly associated with family type, aged 20–40 years, residential location, having 2 children, and retirement status (all *p* < 0.05). In contrast, the High Fertility Intention Group was significantly associated with having no children and with higher depression scores (all *p* < 0.05).

**Conclusions:**

Fertility intention among reproductive-aged women demonstrates significant heterogeneity. This study identified three distinct latent profiles, each characterized by unique patterns of influencing factors. The findings highlight the necessity of moving beyond one-size-fits-all policy approaches and emphasize the importance of developing tailored interventions that account for the specific characteristics and determinants of each subgroup.

## 1 Introduction

With the advancement of modernization, the economic burden of raising children has grown increasingly heavy. Cultural changes have led to a gradual decline in the status of children within families and society. Meanwhile, women's autonomy in fertility decision-making has increased, prompting a reduction in family size (1). This value shift, from prioritizing child quantity to emphasizing child quality, has contributed to a global fertility transition, with countries progressing through distinct stages of this demographic process (2). According to China's National Bureau of Statistics, the country's total population declined by 2.08 million (0.15%) in 2023, reaching 1.409 billion (https://www.usnews.com/news/world/articles/2024-01-16/chinas-population-drops-for-2nd-year-raises-long-term-growth-concerns). However, China has experienced a rapid transition to a low fertility level over a relatively short period. Whether measured by the crude birth rate or the total fertility rate (TFR), China entered the era of low fertility in the early 21st century (3). By the end of 2023, China's total fertility rate had dropped to approximately 1.0, which is far below the critical threshold of 1.5 (4). This declining trend in the fertility rate poses significant challenges to the sustainable supply of labor, accelerates the process of population aging, and exerts profound impacts on population structure, basic livelihood security, and social development (5). To address this issue, China has abolished the one-child policy and implemented a series of fertility-supportive measures. However, the effectiveness of these policies has not yet met expectations.

Fertility intention reflects an individual's expectations and attitudes regarding childbearing over a defined period (6), and includes two key dimensions: fertility timing intention, which relates to the planned schedule of having children, and fertility quantity intention, which refers to the total number of children an individual desires to have (7). Multiple factors at both micro and macro levels influence fertility intention, including individual characteristics (8), family status (9), socioeconomic status (SES), and sociocultural environment (10).

Psychological stress theory posits that perceived stress, which disrupts an individual's psychological equilibrium or exceeds their coping resources can induce anxiety (11). Anxiety, in turn, significantly influences behavior and decision-making, including fertility intentions (12). For instance, research on China's universal two-child policy indicated that childbearing-related stress contributed to notable fertility anxiety among young women, subsequently inhibiting reproductive behavior (13). Moreover, a body of domestic and international literature underscores mental health as a key determinant of fertility intention (14, 15). Factors such as anxiety, depression, identity transition, and future-related concerns may collectively shape individuals’ childbearing plans.

**Figure 1:**
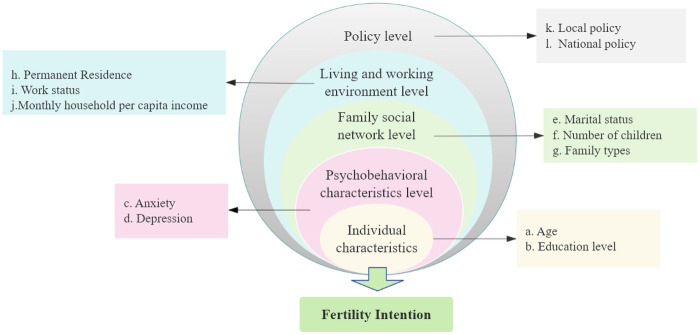
Factors associated with fertility intention based on the socio-ecological model.

Research on factors influencing fertility intention has predominantly focused on women of reproductive age, yet the scope of inquiry remains relatively narrow and lacks a systematic examination of the multifactorial influences shaping their childbearing plans (16, 17). In recent years, fertility research has increasingly incorporated psychosocial perspectives. As highlighted by Neto et al., emotional challenges, relational dynamics, and ethical considerations are central to fertility decision-making (18). However, existing studies have largely concentrated on analyzing single-factor correlates of fertility intention, overlooking individual heterogeneity—specifically, the varied response patterns of reproductive-aged women across multiple fertility-related indicators, which cannot be adequately explained by socioeconomic or demographic characteristics alone. Furthermore, the underlying mechanisms through which psychological and social factors influence different subgroups remain unclear. Therefore, this study adopts the Socio-Ecological Model (SEM) as the guiding framework (Figure 1). This model posits that individual health outcomes arise from the interactions between personal and environmental factors. Its structure comprises five interrelated levels, ranging from the most proximal to the most distal: individual characteristics, psychological and behavioral lifestyle, family and social networks, living and working environments, and policy (19, 20). Building on this framework, the present study employs Latent Profile Analysis (LPA) to identify distinct subtypes of fertility intention and explore their underlying influencing mechanisms. The findings aim to provide an evidence-based foundation for developing more targeted policy interventions. This study is expected to provide empirical evidence for policymakers to formulate effective population policies, thereby addressing the challenges posed by the declining population birth rate.

## 2 Methods

### 2.1 Survey design and participants

Our data were derived from a large-scale cross-sectional survey, the Psychology and Behavior Investigation of Chinese Residents in 2021 (PBICR 2021) [https://www.x-mol.com/groups/pbicr]. This survey was conducted from July 10 to September 15, 2021. The survey employed a multistage sampling method across 31 provinces/autonomous regions/municipalities in mainland China. Using the random number table method, 120 cities were selected, including the capital and two to six prefectural cities in each province/ autonomous region. Based on data from the Seventh National Population Census of China (2021), residents from these 120 cities were selected using quota sampling (quota attributes: sex, age, and urban–rural distribution), ensuring the distributions of these variables in the final sample aligned with population characteristics. Goodness-of-fit tests revealed no significant differences between the sample and the national population across key demographic variables. Given the close alignment of the sample distribution with the national demographic structure after quota adjustment, sampling weights were not applied in the subsequent analysis. We administered 11,709 questionnaires and collected 11,031 valid responses, yielding a response rate of 94.2%.

All participants provided informed consent prior to data collection, and all data were handled with strict confidentiality. This study received ethics approval from the Jinan University Ethics Committee (Approval No. JNUKY-2021-018) and was performed in full adherence to the guidelines and regulations of the Declaration of Helsinki.

The inclusion criteria were as follows: (1) participants held Chinese nationality; (2) participants were women aged 20–49 years who were not currently pregnant. Although China's family planning regulations define women's reproductive age as 15–49 years, the legal age of marriage for women in China is 20 years. Therefore, this study selected data for women aged 20–49 years (21). (3) participants volunteered to participate in the research and completed a consent form; (4) were able to understand the content of each questionnaire item. The exclusion criteria included (1) participants with unconsciousness or severe mental disorders and (2) those unwilling to cooperate or be involved in similar projects. Ultimately, according to the inclusion and exclusion criteria, we obtained a valid sample of 2,973 for this study after deleting data with missing values and outliers.

### 2.2 Survey instruments

#### 2.2.1 General information questionnaire

This included age, education level, marital status, mean monthly household income per capita (CNY), family types, permanent residence, number of children, and work status.

#### 2.2.2 Measures fertility intention

To accurately measure fertility intention, researchers typically use the number of desired children and ideal number of children as indicators of participants’ fertility intention. In this study, fertility intention among reproductive-aged women was comprehensively measured by three questions: “Considering policy and other external constraints, how many children do you wish to have?” and “Do you plan to have a second/third child?” Respondents recorded their responses by moving a slider, which ranged from 0 (No desire) to 5 (Strong desire), with higher scores signifying a higher level of fertility intention.

#### 2.2.3 Patient health questionnaire-9

The Patient Health Questionnaire-9 (PHQ-9) is used to evaluate individuals’ depression symptoms (22). The PHQ-9 consists of 9 items, each scored 0–3, yielding a total score range of 0–27. Scores are interpreted as: 0–4 (no depression), 5–9 (depressive symptoms), and 10–14 (marked depressive symptoms). In this study, Cronbach's *α* of the PHQ-9 was 0.930.

#### 2.2.4 Generalized anxiety disorder-7

Anxiety symptoms over the past two weeks were assessed using the 7-item Generalized Anxiety Disorder-7 (GAD-7) scale (23). This scale consists of 7 items, each rated on a 4-point Likert scale (0 = “Not at all”, 1 = “Several days”, 2 = “More than half the days”, 3 = “Nearly every day”). The total score of the GAD-7 ranges from 0 to 21, with higher scores indicating greater anxiety severity. The Chinese version of the GAD-7 has been validated and has demonstrated strong reliability and validity (24). In this study, Cronbach's α of the GAD-7 was 0.940.

### 2.3 Statistical analysis

Latent Profile Analysis (LPA) was conducted using Mplus 8.3 to identify unobserved subgroups of fertility intention among reproductive-aged women. The number of latent classes was not predetermined. The modeling process started with a single-class model and sequentially increased the number of classes. Model estimation and evaluation were performed iteratively until the optimal solution was achieved. The following fit indices were used for model selection: the Akaike Information Criterion (AIC), the Bayesian Information Criterion (BIC), and the sample size-adjusted BIC (aBIC), with lower values indicating better fit. Classification accuracy was assessed by entropy, ranging from 0 to 1, with values ≥ 0.8 suggesting adequate classification. The Lo-Mendell-Rubin Likelihood Ratio Test (LMRT) and the Bootstrapped Likelihood Ratio Test (BLRT) were used to compare the fit between the k-class and (k-1)-class models, where a significant result (*P* < 0.05) supported the k-class solution. Additionally, the classification probability matrix was examined; diagonal values greater than 0.7 indicated good classification reliability.

Following the identification of the optimal latent profile model, subsequent analyses were conducted using SPSS 27.0. Continuous variables that followed a normal distribution were presented as mean ± standard deviation (SD), while categorical variables were presented as frequency (percentage) [*n* %)]. Group comparisons were performed using the chi-square test (or Fisher's exact test when expected cell counts were <5) for categorical variables and one-way analysis of variance (ANOVA) for normally distributed continuous variables. Variable selection for multivariable analysis was based on both bivariate associations and theoretical relevance within the Socio-ecological Model (SEM) framework. Therefore, all variables that were either statistically significant in bivariate analyses (*p* < 0.05) or deemed theoretically important for fertility intention were retained as candidates for the final model. A multinomial logistic regression model was then constructed with the latent fertility intention profiles as the dependent variable to identify factors independently associated with profile membership. A two-tailed *p*-value < 0.05 was considered statistically significant in all analyses. In the present study, latent class models ranging from 1 to 4 classes were sequentially fitted to the fertility intention scores of reproductive-aged women. The model fit indices for each profile are presented in Table 1.

**Table 1:** Fit indices for latent classes of fertility intention among women of reproductive age.

Model	AIC	BIC	aBIC	Entropy	LMRT	BLRT	Category probability(%)
1	26,411.847	26,447.831	26,428.767	–	–	–	1.000
2	24,091.110	24,151.083	24,119.309	0.818	<0.001	<0.001	0.496/0.504
3	23,251.186	23,335.149	23,290.665	0.861	<0.001	<0.001	0.251/0.513/0.236
4	22,443.302	22,551.254	22,494.061	0.862	0.219	0.226	0.492/0.192/0.267/0.049

## 3 Results

### 3.1 Individual characteristics

This study enrolled 2,973 women of childbearing age, of whom 2,262 (76.1%) were urban residents and 711 (23.9%) were rural residents. The 20–30 age group constituted a substantial proportion of the sample, while the majority of families had a per capita monthly income ranging between 3,001 and 6,000 yuan. Additional demographic details are presented in Table 2. Table 2 examined associations between demographic, socioeconomic, psychological factors and latent fertility intention classes via chi-square test, Fisher's exact test, and ANOVA. Results showed no significant differences in educational level (*χ*² = 8.302, *p* = 0.405) and monthly household per capita income (*χ*² = 14.448, *p* = 0.273), while other factors (age, marital status, family type, etc.) differed significantly across the three classes (all *p* < 0.001).

**Table 2:** Univariate analysis of latent classes of fertility intention Among women of reproductive Age.

Characteristics	*N* (%)	Fertility intention decline group *n* = 25.1%	Low Fertility Intention Group *n* = 51.3%	High fertility intention group *n* = 23.6%	Statistic	*p*
Age						
20–30	1,400 (47.1)	317	751	332	*χ*² = 36.957^a^	<0.001***
31–40	687 (23.1)	217	292	178		
41–49	886 (29.8)	213	481	192		
Education level						
Junior high school and below	113 (3.8)	27	47	39	χ² = 8.302^a^	0.405
Technical secondary/high school	701 (23.6)	203	326	172		
University and above	2,159 (71.6)	517	1,151	491		
Marital status						
Unmarried	1,197 (40.3)	246	665	286	χ² = 100.264^a^	<0.001***
Have a spouse	1,701 (57.2)	486	813	402		
Divorced	75 (2.5)	15	46	14		
Monthly household per capita income (CNY)						
≤3,000	857 (28.8)	227	405	225	χ² = 14.448^a^	0.273
3,001–6,000	1,172 (39.4)	311	607	254		
6,001–9,000	506 (17.0)	116	281	109		
≥90,001	438 (14.7)	93	231	114		
Family types						
Childless couple households	497 (16.7)	112	243	142	χ² = 75.944^a^	<0.001***
Nuclear family	1,938 (65.2)	495	1,008	435		
Stem family	232 (7.8)	80	92	60		
Joint family	75 (2.5)	20	28	27		
Single-parent family	231 (7.8)	40	153	38		
Permanent residence						
Urban	2,262 (76.1)	538	1,224	500	χ² = 22.795^a^	<0.001***
Rural	711 (23.9)	209	300	202		
Number of children					χ² = 87.023^a^	<0.001***
0	1,461 (49.1)	300	821	340		
1	933 (31.4)	183	592	158		
2	512 (17.2)	257	100	155		
≥3	67 (2.3)	7	11	49		
Work status					29.317^b^	<0.001***
No job	555 (18.7)	163	246	146		
Retired	30 (1.0)	1	22	7		
Student	831 (28.0)	174	458	199		
Full-time	1,557 (52.4)	409	798	350		
Anxiety (SD)	4.78 ± 4.595	4.16 ± 3.847	4.36 ± 4.301	6.37 ± 5.499	*F* = 57.213^c^	<0.001***
Depression (SD)	6.64 ± 5.474	5.75 ± 4.385	6.20 ± 5.006	8.53 ± 6.880	*F* = 58.493^c^	<0.001***

^a^ Chi-square test.

^b^ Fisher's exact test.

^c^ Analysis of variance (ANOVA).

Statistical significance was set at:

*p < 0.05.

**p < 0.01.

*** *p* < 0.001.

### 3.2 Latent profile analysis of fertility intention in women of childbearing age

In this study, we sequentially constructed latent class models with 1–4 classes to fit the fertility intention scores of reproductive-aged women, and the corresponding model fit indices are summarized in Table 1. As the number of classes increased, the AIC, BIC, and aBIC values consistently decreased, reaching their minimum in the 4-class model. For both the 2-class and 3-class models, entropy values exceeded 0.8, with negligible differences between them. The LMRT and the BLRT results for these two models were statistically significant (both *p* < 0.05). In contrast, neither the LMRT nor the BLRT reached statistical significance for the 4-class model (both *p* < 0.05).

Further model selection was guided by interpretability and clinical relevance. In the 4-class model, certain subgroups comprised a relatively small proportion of participants. Excessive partitioning may lead to fragmented classification with limited practical applicability, and therefore this model was not retained. Although the 2-class model demonstrated statistically significant LMRT and BLRT results, its AIC, BIC, and aBIC values remained higher than those of models with more classes, and the two resulting subgroups showed a markedly uneven distribution of participants, which diminished the utility of the classification for targeted intervention. In contrast, the 3-class model exhibited improved model fit indices compared to the 1-class and 2-class models, along with reasonable subgroup sizes and clearer interpretability. Thus, based on a comprehensive evaluation of statistical fit, classification accuracy, and practical relevance, the 3-class model was selected as the optimal solution for capturing latent profiles of fertility intention among reproductive-aged women.

**Figure 2:**
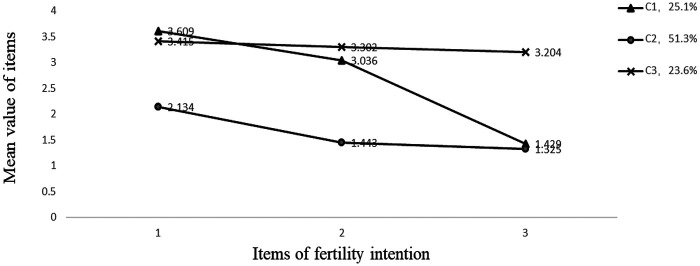
Latent profile model of fertility intention among women of reproductive age.

Based on the optimal three-class model, the latent profile distributions of the three fertility intention items among reproductive-aged women are illustrated in Figure 2. Class 1 (*n* = 746, 25.1%) was characterized by low overall scores and a descending trajectory across items, reflecting a gradually decreasing fertility intention; this group was labeled the “Fertility Intention Decline Group.” Class 2 (*n* = 1,526, 51.3%) displayed moderate mean scores relative to the other two classes and was designated the “Low Fertility Intention Group.” Class 3 (*n* = 702, 23.6%) consistently demonstrated the highest mean scores across all items and was therefore identified as the “High Fertility Intention Group.”

### 3.3 A univariate analysis of potential categories of depression in women of childbearing age

Univariate analysis results indicated that there were statistically significant differences in factors influencing fertility intention fertility intention among reproductive-aged women across different latent classes (Table 2). Except for educational level and monthly household income (all *p* > 0.05), all other factors showed statistically significant differences between the classes (all *p* < 0.05).

### 3.4 Multivariate analysis of potential profiles of depression in women of childbearing age

A multinomial logistic regression was performed to identify factors associated with latent fertility intention subgroups. The dependent variable was the three-class fertility intention profile (1 = Fertility Intention Decline Group, 2 = Low Fertility Intention Group, 3 = High Fertility Intention Group). Variables that exhibited statistically significant differences in the univariate analyses (see Table 3 for definitions) were entered as independent variables, with the Fertility Intention Decline Group serving as the reference category.

**Table 3:** Variable assignment criteria.

Variable	Assignment mode
Age	20–30 = 1; 31–40 = 2; 41–49 = 3
Family types	Childless couple households nuclear family = 1; stem family = 2; Joint family = 3; single-parent family = 4
Permanent residence	Urban = 1; Rural = 2
Quantity of children	0 = 0; 1 = 1; 2 = 2; 3 = 3
Work status	No job = 1; retired = 2; student = 3; full-time = 4
Education level	Junior high school and below = 1; technical secondary/high school = 2; University and above = 3
Marital status	Unmarried = 1; have a spouse = 2; divorced = 3
Monthly household per capita income (Yuan)	≤3,000 = 1; 3,001–6,000 = 2; 6,001–9,000 = 3; ≥9,001 = 4
Anxiety	Measured value
Depression	Measured value

The results revealed that, compared to the Fertility Intention Decline Group, reproductive-aged women who were part of spousal or nuclear families, aged 20–40 years, resided in urban areas, had two children, or were retired showed a significantly higher likelihood of belonging to the Low Fertility Intention Group (all *p* < 0.05). Conversely, with no children, or with higher depression scores were significantly more likely to be classified into the High Fertility Intention Group relative to the reference group [(all *p* < 0.05); see Table 4].

**Table 4:** Multivariate logistic regression analysis of different latent profiles of fertility intention Among women of reproductive Age.

Dependent variable	Independent variable	*b*	*SE*	*Wald* χ² value	*P value*	*OR* (95%CI)
The Low Fertility Intention Group	Constant	−1.452	0.619	5.505	0.019	–
	Family type					
	Childless couple households	−0.485	0.238	4.146	0.042*	0.616 (0.386–0.982)
	Nuclear family	−0.481	0.207	5.394	0.020*	0.618 (0.412–0.928)
	Age					
	1	−0.943	0.182	26.790	<0.001***	0.389 (0.273–0.557)
	2	−0.874	0.143	37.248	<0.001***	0.417 (0.315–0.553)
	Place of residence					
	1	0.311	0.114	7.411	0.006**	1.365 (1.091–1.707)
	Number of children					
	2	−1.361	0.504	7.286	0.007**	0.256 (0.095–0.689)
	Permanent residence					8.337 (1.058–65.685)
	Retired	2.121	1.053	4.055	0.044*	
	Anxiety	−0.015	0.021	0.530	0.985	1.333 (0.946–1.026)
	Depression	0.032	0.017	3.466	0.063	1.033 (0.998–1.069)
The high fertility intention group	Constant	1.432	0.602	5.657	0.017*	–
	Number of children					
	0	−1.822	0.468	15.166	<0.001**	0.162 (0.065–0.405)
	Anxiety	0.037	0.023	2.635	0.105	1.038 (0.992–1.085)
	Depression	0.063	0.019	10.635	0.001**	1.065 (1.025–1.106)

This model was adjusted for age, family type, permanent residence, number of children, work status, education level, marital status, monthly household per capita income (CNY), anxiety, and depression.

Statistical significance was set at:

* *p* < 0.05.

** *p* < 0.01.

*** *p* < 0.001.

## 4 Discussion

### 4.1 Potential profile characteristics of fertility intention in women of childbearing age

Three distinct fertility intention profiles were identified among reproductive-aged women: a Fertility Intention Decline Group (25.1%), a Low Fertility Intention Group (51.3%), and a High Fertility Intention Group (23.6%). These findings confirm substantial heterogeneity in fertility intention, addressing a key limitation of conventional variable-centered approaches that often overlook within-population differences. Accordingly, this study offers a person-centered perspective that advances the understanding of the complex structure of fertility intention in this population.

The Fertility Intention Decline Group (25.1%) was characterized by a sharp decrease in scores across fertility intention items. Although this group reported a relatively high initial overall fertility intention, their willingness to have a second or third child declined markedly. This discrepancy may be attributable to the combined physical and psychological transitions associated with childbirth, as well as the multifactorial nature of fertility decisions, which often encompass economic pressures, perceived constraints on personal autonomy, and persistent work-family conflict (25). When these cumulative stressors exceed an individual's coping capacity, the positive affective rewards of parenting may be overshadowed, heightening emotional distress and further dampening the intention for subsequent children (26). Consequently, women within this profile may engage in a reassessment of their childbearing plans (27).

The Low Fertility Intention Group comprised 51.3% of the sample, showing no significant within-group differences across the three fertility intention items, yet its scores were consistently and significantly lower than those of the other two groups. As this group constitutes over half of the total participants, it reflects a prevalent pattern of low fertility intention. This finding is consistent with those of Zhao et al. (28), who also reported low overall fertility intention among reproductive-aged women in China. Potential reasons include the following: With the rapid development of the internet, women of reproductive age raised in a multicultural context are exposed to diverse fertility values. They no longer adhere to the traditional marital concept of their parents that “marriage and having children are a matter of course”; instead, they are more focused on self-development and personal autonomy (29). Furthermore, child-rearing entails substantial investment of time, energy, and financial resources, often at the cost of sleep, leisure, social engagement, and individual growth after childbirth.

The High Fertility Intention Group (23.6%) exhibited the strongest overall fertility intention, yet also displayed the highest level of depression. This contradictory finding differs from some previous studies (14), which typically associated poorer mental health with lower fertility intention. An examination of underlying mechanisms suggests that the depressive symptoms observed in this group may not stem directly from childbearing *per se*, but rather from a perceived “expectation-support mismatch”: women in this profile often endorse strong ideals of family completeness while simultaneously recognizing the substantial investments required for raising multiple children, including time demands, economic pressures, and limited social support. Furthermore, societal expectations surrounding the role of “mothers of multiple children”—such as the pressure to “balance work and family”—likely compound their psychological burden. These findings highlight the importance of attending to the mental well-being of women who intend to have more than one child.

### 4.2 Influencing factors of potential characteristics of depression in women of childbearing age

#### 4.2.1 Individual characteristics

##### 4.2.1.1 Age

This study found that fertility intention was higher among women aged 20–40 years. This observation aligns with prior work by Dong et al. (30), which highlighted that advancing age is associated with increased physiological depletion and elevated health risks related to childbirth. Age thus functions as a key determinant of reproductive capacity: older women face greater reproductive challenges and higher perinatal risks, such as an increased likelihood of delivering infants with congenital anomalies. As women grow older, declines in personal physical health, stamina, and general energy are often accompanied by the aging of their own parents, whose diminished health status further limits their ability to provide childcare support. Collectively, these factors contribute to a reduction in fertility intention with advancing age.

#### 4.2.2 Family social network level

##### 4.2.2.1 Family type

The core characteristic of dual-career couples (childless) is the absence of children; these families consist solely of spouses. Egalitarian dynamics within these partnerships—characterized by equitable gender roles and shared decision-making—may weaken traditional family norms centered on “reproductive lineage continuity.” Research has indicated that couples who endorse egalitarian values tend to prioritize personal development over childbearing, and their “risk perceptions” concerning marriage (e.g., threats to relationship stability or career conflicts) can further lower fertility preferences (31). Moreover, another study found that when couples face fewer external social constraints in partner selection, their ideal number of children decreases significantly (*β* = −0.21, *p* < 0.01) (32).

The nuclear family, defined by the structure of a married couple living with their unmarried children, represents the predominant family model in contemporary society. Such families often concentrate their economic and temporal resources on the education and development of existing children, thereby substantially elevating the opportunity cost associated with having additional children. A study found that in nuclear families, educational expenses for the first child account for 30%–40% of household disposable income, a factor directly associated with reduced parental intention for a second child (33).

##### 4.2.2.2 Number of children

The present study also observed that the number of existing children influences fertility intention among reproductive-aged women. The intention to have a second child was stronger among women with one child than the intention to have a third child was among women with two children. In contemporary societies that emphasize gender equality, mothers are increasingly expected to maintain career involvement, which limits the time available for childcare. In the absence of external support, this constraint substantially reduces fertility intention. Moreover, parents assume full responsibility for risks related to their children's health and education. The rising standards of modern parenting—such as the emphasis on quality-focused education and greater attention to mental well-being—have further heightened such risk perceptions. Studies have shown a significant negative correlation between parents’ concerns about their “children's future development” and their fertility intention (r = −0.32, *p* < 0.001) (34).

#### 4.2.3 Living and working environment level

##### 4.2.3.1 Permanent residence

Hukou (household registration) differences also exert an impact on fertility intention. At the institutional constraint level, hukou status directly determines eligibility for fertility-related public services and policy support: while urban hukou holders have access to higher-quality medical and educational resources, they face higher “institutional thresholds” for childbearing, such as competition for school district housing and an imbalance between childcare expenditures and fertility subsidies, which significantly inhibits their fertility intention (35). In contrast, migrant populations without local hukou—due to the separation between their hukou registration and place of residence—struggle to equally access fertility support in their destination areas, including free pre-pregnancy examinations and compulsory education for children. Additionally, they confront time costs associated with returning to their hometowns for childbirth and reimbursement barriers for medical treatment in non-hukou locations, all of which affect their fertility intention (36).

##### 4.2.3.2 Work status

Focusing on employed women, studies have found that career engagement itself significantly reduces fertility intention (37). While improvements in occupational quality may enhance the capacity to withstand financial risks, the accompanying rise in opportunity costs—such as potential career interruptions due to childbearing—can conversely suppress fertility intention (38). It is also important to note that disparities in occupational quality contribute to varying levels of resilience to fertility-related risks across socioeconomic groups. Higher socioeconomic status generally confers greater ability to buffer the adverse effects of economic uncertainty on fertility plans. After retirement, reduced personal income can further constrain fertility decisions. Research indicates that pressures related to child-rearing expenses, familial expectations, and marital relationship maintenance collectively suppress the fertility intention of employed women through multiple pathways (39).

#### 4.2.4 Psychobehavioral characteristics level

##### 4.2.4.1 Anxiety

This study found no significant association between anxiety and fertility intention, which is inconsistent with previous research (14). This discrepancy may be attributed to three moderating effects: first, “heterogeneity in the type of anxiety”—personal health anxiety and career anxiety may inhibit fertility intention, whereas social anxiety stemming from family/societal “marriage and fertility pressure” may actually increase fertility intention. This heterogeneity suggests that future research should distinguish sources of anxiety to more precisely understand their effects. Second, the moderating role of socio-cultural factors—the traditional Chinese family value that “childbearing is a life responsibility” may counteract the negative impact of anxiety on fertility intention. Third, this study did not include moderating variables such as social support and coping strategies, which could mitigate the inhibitory effect of anxiety on fertility intention (40). For example, a study on China's universal two-child policy found that fertility-related stress led to significant fertility anxiety among young women, thereby suppressing their childbearing behavior (13). Another study on third-child fertility decisions indicated that fertility-related and parenting-related anxiety were negatively correlated with fertility intention (14). These findings suggest that anxiety may play a mediating role between stress and fertility intention, a hypothesis that could be tested in future research. Finally, the measurement of variables focused on state anxiety rather than trait anxiety, which may not fully capture the long-term anxiety tendencies relevant to fertility intention. Additionally, certain demographic variables (e.g., marital status, economic income) may interact with anxiety, further attenuating the main effect of anxiety.

##### 4.2.4.2 Depression

In contrast, depression showed a significant negative correlation with fertility intention: the more severe the depressive symptoms, the lower the intention to have a second or third child—except in the high fertility intention group, where depression likely stemmed from the conflict between fertility aspirations and perceived inadequate support. Individuals with persistent depressive symptoms often experience impaired information-processing capacity and diminished stress-management abilities (41). As a psychologically vulnerable group, those with depressive symptoms are frequently accompanied by emotional distress and negative affect. For example, women with depressive symptoms typically perceive weaker partner support, which may reduce their satisfaction with current family well-being, exacerbate concerns and anxiety about future parenting responsibilities, and ultimately weaken their fertility intention (42). Similarly, lower “family socioeconomic status” may indirectly suppress fertility intention by inducing feelings of future uncertainty and depressive emotions. In this study, the co-occurrence of high fertility intention and high depression levels in one group may precisely reflect the conflict between “high fertility aspirations” and “perceived real-world barriers (e.g., childcare costs, insufficient social support).” Furthermore, individuals with depression often exhibit heightened risk aversion, a psychological trait that can lead to behavioral withdrawal (43). Such withdrawal may serve as a protective mechanism for depressed individuals, helping them avoid potential adverse situations—for example, choosing to have fewer or no children to evade heavy financial burdens and negative emotions associated with inadequate parenting.

#### 4.2.5 Policy level

Based on the characteristics and influencing factors of the three fertility intention subtypes, it is necessary to construct a “precision-oriented and multi-level” fertility support system to avoid a one-size-fits-all policy approach:

For the Low Fertility Intention Group (representing the largest proportion), the key lies in reducing childbearing costs and alleviating work–family conflict. Economically, measures should include expanding the coverage of childbearing allowances, extending maternity leave, and reducing personal income tax. Housing subsidies and educational expense reductions should be provided for multi-child families, while ensuring job stability for reproductive-age women. A tiered childcare subsidy system could be implemented: for families with 1–2 children, monthly cash subsidies should be provided based on regional economic levels until the child reaches 6 years of age (primary school entry); for families with three children, the monthly subsidy should be increased to offset the marginal costs associated with additional children. In terms of services, there should be an expansion in the supply of accessible and affordable childcare, the introduction of flexible working arrangements, an extension of paternity leave, and encouragement of more balanced sharing of childcare responsibilities between spouses. On the conceptual level, science-based parenting knowledge should be disseminated through multimedia platforms to help alleviate “parenting anxiety” (44).

For the Fertility Intention Decline Group, the primary focus should be on addressing the realistic dilemma of “being willing yet hesitant to have children.” This involves establishing a comprehensive parenting support service system, including provision of parenting skill training and postpartum psychological counselling services. Simultaneously, public services related to childbirth should be optimized by simplifying the procedures for birth and household registration. For instance, a “one-stop service” integrating prenatal check-ups, delivery, and birth registration could be implemented, allowing parents to complete procedures via online platforms or government service counters. Additionally, the government should provide one-time childbearing incentives for second and third children to alleviate the financial pressure on multi-child families.

For the High Fertility Intention Group, the primary focus should be on providing mental health support and enhancing social support systems. Integrate preconception mental health screening into routine maternal and child health services using standardized tools such as the PHQ-9 and GAD-7 scales, particularly for women planning multiple births; those identified with moderate to severe depressive or anxiety symptoms should be referred to specialized psychological counseling services. Additionally, an official “Fertility Psychological Counseling Hotline” could be established to provide accessible emotional support for women of reproductive age. Concurrently, community-based parenting mutual aid platforms should be developed to encourage family members and neighbors to participate in child care, thereby alleviating parenting stress. Experienced women could be recruited as group leaders to organize regular online or offline support meetings each month, focusing on emotional sharing and coping strategy exchange. These group leaders should also receive basic psychological first-aid training to help identify and support women at heightened risk of depression.

### 4.3 Cultural influences

In traditional Chinese society, procreation was culturally mandated to ensure lineage continuity, encapsulated in the saying “having no descendants is the greatest unfilial act.” This belief was central to social identity and closely tied to Confucian filial piety, where bearing children was a key moral duty (45). Since the 1980s, however, family-planning policies and rising costs of child-rearing have weakened traditional norms such as “more children, more blessings,” contributing to today's low fertility. Modern cultural shifts further suppress fertility intention. For many women, childbearing is now a matter of personal choice and gender equality rather than familial obligation. Concepts like “DINK” (double-income, no kids) and “fewer but better-raised children” have reduced desired family size. Finally, emerging social norms also play a role. “Parenting involution”—intense competition over children's education and development—has turned parenting into a source of economic and psychological strain. The anxiety of “not raising children well” is a major concern for those with low or declining fertility intention (44). Although direct evidence is still limited, studies suggest that perceived involution can lower fertility intention. From a resource-scarcity perspective, when individuals perceive limited social and economic resources, they tend to make cautious, often restrictive decisions about having children (46).

## 5 Limitations

This study has several limitations. First, reliance on cross-sectional data from 2021 restricts the ability to infer causal relationships between the identified factors and fertility intentions. Fertility intentions can be influenced by marital duration (47), and may shift over time in response to policy changes (48), Future studies should adopt longitudinal designs to track the dynamic nature of fertility intentions. Future studies should adopt longitudinal designs to track the dynamic nature of fertility intentions. Second, fertility intention was measured with only three self-reported items, which may not fully capture related dimensions such as intended timing or motivation for childbearing. Subsequent research could employ validated multi-item scales to improve measurement accuracy. Third, key variables such as social support, coping strategies, and regional policies were not included, possibly affecting results. Fourth, the sample only included mainland China, so cross-cultural studies are needed to test generalizability.

## 6 Conclusions

Fertility intention among women of reproductive age can be categorized into three subtypes: the Fertility Intention Decline Group, the Low Fertility Intention Group, and the High Fertility Intention Group. The study finds significant differences across these subtypes in terms of family type, age, place of residence, number of children, employment status, and depression. Therefore, to enhance the fertility intention of women in the low or declining intention groups, targeted interventions should include not only fostering positive attitudes toward childbearing but also optimizing public health and service environments, and constructing a family-friendly fertility support system. Additionally, attention should be paid to the well-being of women planning to have a second or third child, with the development of tailored policies.

## Data Availability

The datasets presented in this study can be found in online repositories. The names of the repository/repositories and accession number(s) can be found below: https://www.x-mol.com/groups/pbicr.
